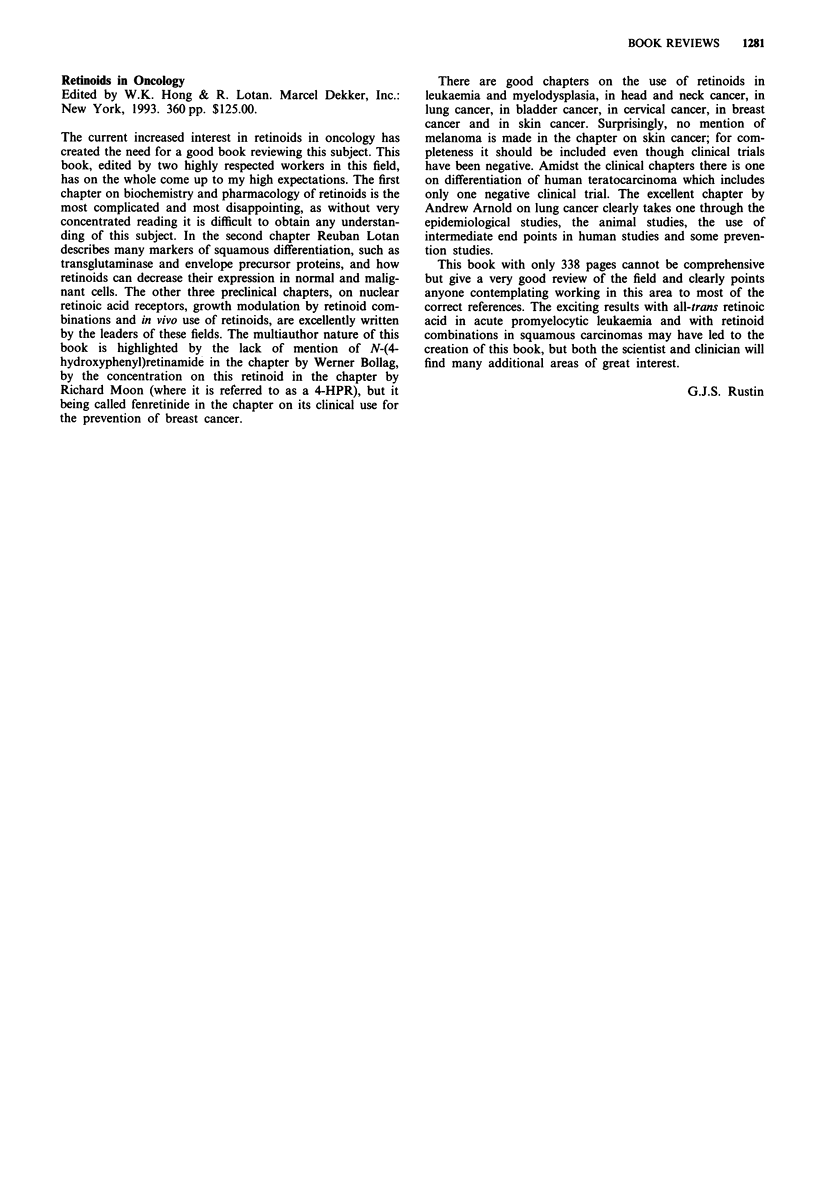# Retinoids in Oncology

**Published:** 1994-12

**Authors:** G.J.S. Rustin


**Retinoids in Oncology**
Edited by W.K. Hong & R. Lotan. Marcel Dekker, Inc.: New York, 1993. 360 pp. $125.00.

The current increased interest in retinoids in oncology has created the need for a good book reviewing this subject. This book, edited by two highly respected workers in this field, has on the whole come up to my high expectations. The first chapter on biochemistry and pharmacology of retinoids is the most complicated and most disappointing, as without very concentrated reading it is difficult to obtain any understanding of this subject. In the second chapter Reuban Lotan describes many markers of squamous differentiation, such as transglutaminase and envelope precursor proteins, and how retinoids can decrease their expression in normal and malignant cells. The other three preclinical chapters, on nuclear retinoic acid receptors, growth modulation by retinoid combinations and *in vivo* use of retinoids, are excellently written by the leaders of these fields. The multiauthor nature of this book is highlighted by the lack of mention of *N*-(4-hydroxyphenyl)retinamide in the chapter by Werner Bollag, by the concentration on this retinoid in the chapter by Richard Moon (where it is referred to as a 4-HPR), but it being called fenretinide in the chapter on its clinical use for the prevention of breast cancer.

There are good chapters on the use of retinoids in leukaemia and myelodysplasia, in head and neck cancer, in lung cancer, in bladder cancer, in cervical cancer, in breast cancer and in skin cancer. Surprisingly, no mention of melanoma is made in the chapter on skin cancer; for completeness it should be included even though clinical trials have been negative. Amidst the clinical chapters there is one on differentiation of human teratocarcinoma which includes only one negative clinical trial. The excellent chapter by Andrew Arnold on lung cancer clearly takes one through the epidemiological studies, the animal studies, the use of intermediate end points in human studies and some prevention studies.

This book with only 338 pages cannot be comprehensive but give a very good review of the field and clearly points anyone contemplating working in this area to most of the correct references. The exciting results with all-*trans* retinoic acid in acute promyelocytic leukaemia and with retinoid combinations in squamous carcinomas may have led to the creation of this book, but both the scientist and clinician will find many additional areas of great interest.

G.J.S. Rustin